# Combination antifungal therapy without craniotomy in an immunocompromised patient with rhino-orbito-cerebral mucormycosis: A case report 

**DOI:** 10.22088/cjim.11.2.227

**Published:** 2020

**Authors:** Mohammadreza Salehi, Farhad Shahi, Fatema Sadaat Rizvi, Sara Ghaderkhani, Hamed Zainaldain, Sadegh Khodavaisy, Saeed Reza Jamali-Moghaddam, Seyed Ali Dehghan Manshadi, Omid Rezahosseini

**Affiliations:** 1 Imam Khomeini Complex Hospital, Department of Infectious Diseases, Tehran University of Medical Sciences, Tehran, Iran; 2 Department of Adult Hematology and Oncology, Imam Khomeini Complex Hospital, Tehran University of Medical Sciences, Tehran, Iran; 3 School of Medicine, Tehran University of Medical Sciences, Tehran, Iran; 4 Department of Medical Mycology and Parasitology, School of Public Health, Tehran University of Medical Sciences, Tehran, Iran; 5 Ziaeian Hospital, Department of Infectious Diseases, Tehran University of Medical Sciences, Tehran, Iran; 6 Viro-immunology Research Unit, Department of Infectious Diseases 8632, Rigshospitalet, University of Copenhagen, Copenhagen, Denmark

**Keywords:** Mucormycosis, Invasive fungal infection, Combination drug therapies, Liposomal amphotericin B, Posaconazole

## Abstract

**Background:**

Mucormycosis is an uncommon fungal infection caused by the members of the order Mucorales. In susceptible patients, mucormycosis can infect any tissue or organ, and without suitable treatment (i.e., debridement and antifungal therapy), this infection can be fatal. Our patient was a woman with lymphoma and cerebral mucormycosis who was treated with antifungals and without any neurosurgical debridement.

**Case Presentation:**

Herein, we present the case of a 35-year-old woman with diagnosis of B-cell lymphoma and rhino-orbito-cerebral mucormycosis (ROCM). She was a candidate for enucleation of the left eye, orbital decompression, and sinocerebral debridement. Nevertheless, the patient refused eye enucleation and craniotomy. Finally, she was treated with a combination of antifungals and sinus debridement without eye enucleation and craniotomy.

**Conclusion:**

debridement, along with a combination of liposomal amphotericin B (LAMB) and posaconazole, may be a suitable therapeutic option for patients with ROCM, who are not eligible candidates for extensive surgery or craniotomy.

Mucormycosis is an uncommon fungal infection caused by the members of the order Mucorales (1, 2). Sporangiospores are ubiquitously found in decaying organic materials and soil. Mucormycosis mostly transmits to humans via inhalation of sporangiospores, and occasionally by traumatic inoculation (3). Mucorales may also be found in the air and water of healthcare settings and tongue depressors; they are also found in the nose and mouth of healthy individuals (4). The main risk factors for mucormycosis include immunosuppressive and chemotherapy agents, poorly controlled diabetes mellitus, hematologic malignancies (e.g., leukemia and lymphoma), hematopoietic stem cell or solid organ transplantation, and some other factors (1, 5). Although Mucorales can infect any tissue, rhino-orbito-cerebral mucormycosis (ROCM), cutaneous mucormycosis, and pulmonary mucormycosis are more commonly reported. The common presentations of ROCM include perinasal cellulitis, facial pain and paresthesias, headache, lethargy, visual loss, proptosis, and/or palatal ulcer (2, 6). The mortality rate of patients with ROCM, even with treatment, is more than 60% (5). Therefore, prompt debridement of necrotic tissues, systemic administration of antifungal agents, dose reduction of immunosuppressants, and control of underlying diseases can be life-saving (1). Lipid formulations of amphotericin B are the first-line antifungal agents for the treatment of ROCM. 

However, there has been a growing interest in new azoles, such as posaconazole and isavuconazole in recent years. Most of our knowledge about the treatment of mucormycosis is based on the findings of case reports (5). Therefore, experiences and rare cases of this disease are worth reporting. Herein, we present a patient with ROCM, who was treated with a combination of antifungals, i.e., liposomal amphotericin B (LAMB) plus posaconazole, and sinus debridement without craniotomy. The aim of this case report was to demonstrate the clinical course of this patient and increase the current awareness of the efficiency of combination therapy for patients with ROCM, who refuse surgery.

## Case presentation

The patient was a 35-year-old woman with a confirmed diagnosis of high-grade B-cell lymphoma under rituximab, cyclophosphamide, doxorubicin, vincristine, and prednisone (R-CHOP) and cyclophosphamide, vincristine, doxorubicin, high-dose methotrexate/ifosfamide, etoposide, and high-dose cytarabine (CODOX-M/IVAC) regimens. In the final phase of chemotherapy, an infectious disease consultation was requested because of fever (axillary temperature, 38.9°C) and neutropenia (absolute neutrophil count, 200 cell/mL).

**Figure 1:**
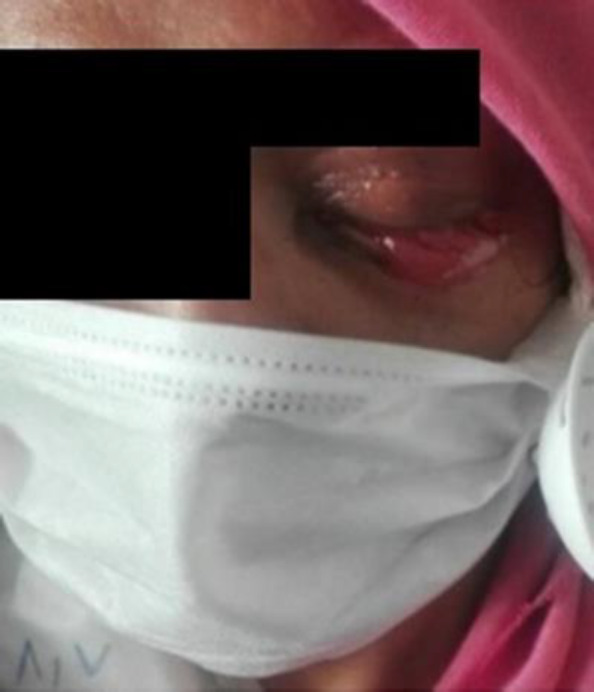
The swollen erythematous left eyelid

On the physical examination, the patient’s left eyelid was swollen and erythematous. She also had headaches and blurred vision (figure 1).

**Figure 2:**
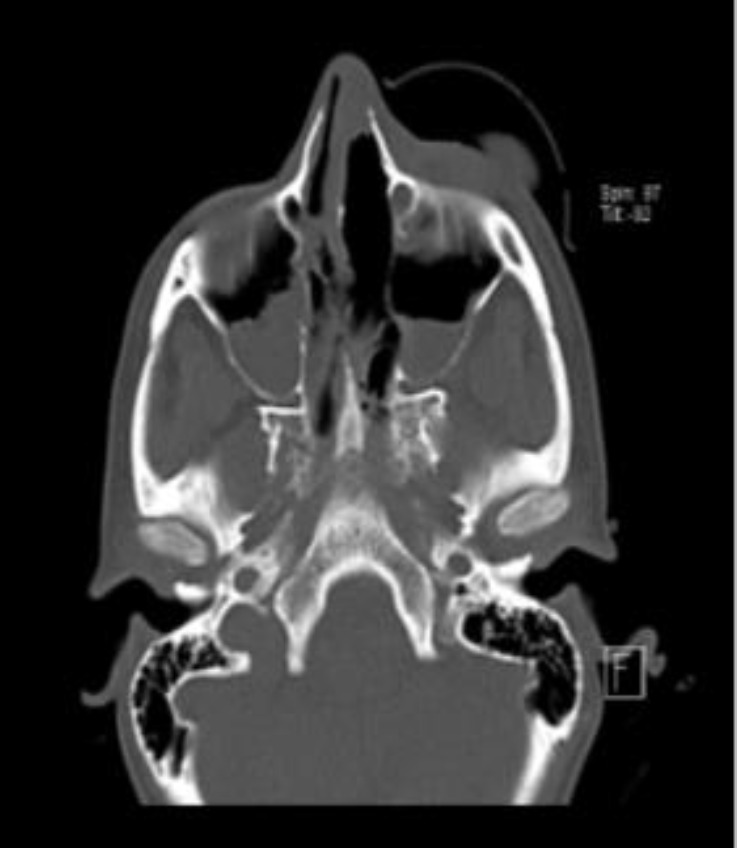
The PNS CT scan (axial view-without contrast) shows a mucosal thickening in both maxillary sinuses

**Figure 3:**
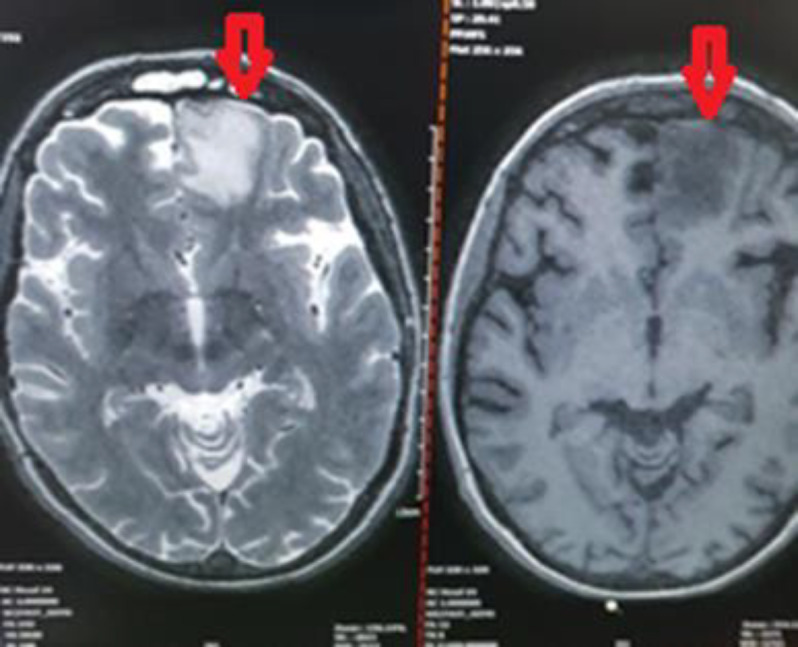
The brain MRI image (MRI-T2 flair view) of an intracranial hyper-intense lesion with dimensions of 20×20 mm in the left frontal lobe (Red arrow)

Since opportunistic infections were highly probable, chemotherapy was immediately stopped, and broad-spectrum antibiotics, including meropenem (2 g q8h), vancomycin (1 g q12h), and LAMB (300 mg/daily), were prescribed. In addition, paranasal sinus (PNS) CT scan and brain magnetic resonance imaging (MRI) were requested. The PNS CT scan showed mucosal thickening in both maxillary sinuses (figure 2). The brain MRI images revealed an intracranial lesion (about 20×20 mm) in the left frontal cortex (MRI-T2 flair view) (figure 3). Her laboratory tests revealed C-reactive protein (CRP): +++, erythrocyte sedimentation rate (ESR): 83 mm/h, galactomannan: 0.3 and blood cultures: negative.

**Figure 4:**
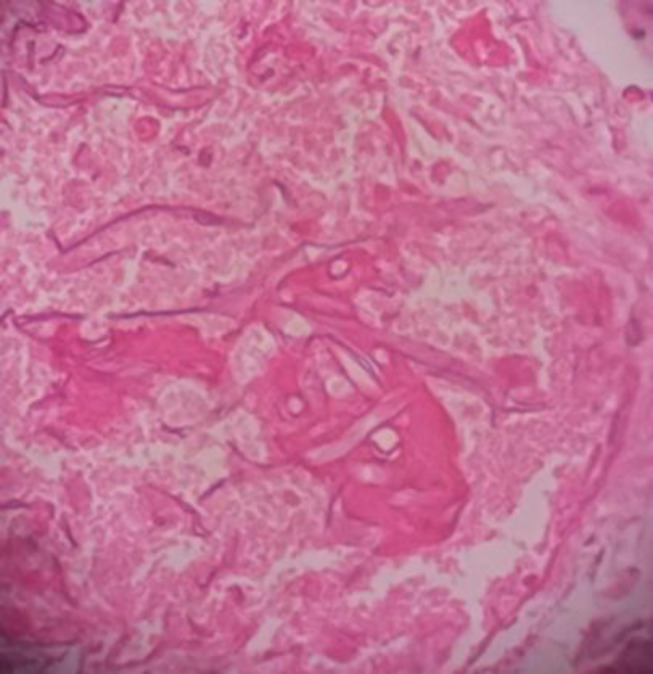
The histological examination of sinus debridement (aseptate ribbon-like hyphae)

The findings were compatible with ROCM; accordingly, sinus endoscopic biopsy and debridement was carried out, and the specimens were sent to the laboratory. Ribbon-like hyphae, compatible with mucormycosis, were reported in the histopathological examination (figure 4). Considering the extent of ROCM, the patient was a candidate for enucleation of the left eye, orbital decompression, and sinocerebral debridement. Nevertheless, the patient refused eye enucleation and craniotomy. Therefore, multiple endoscopic sinus debridement procedures were carried out, and posaconazole (200 mg /Q 6 hours) was added to LAMB as a combination antifungal treatment. 

**Figure 5:**
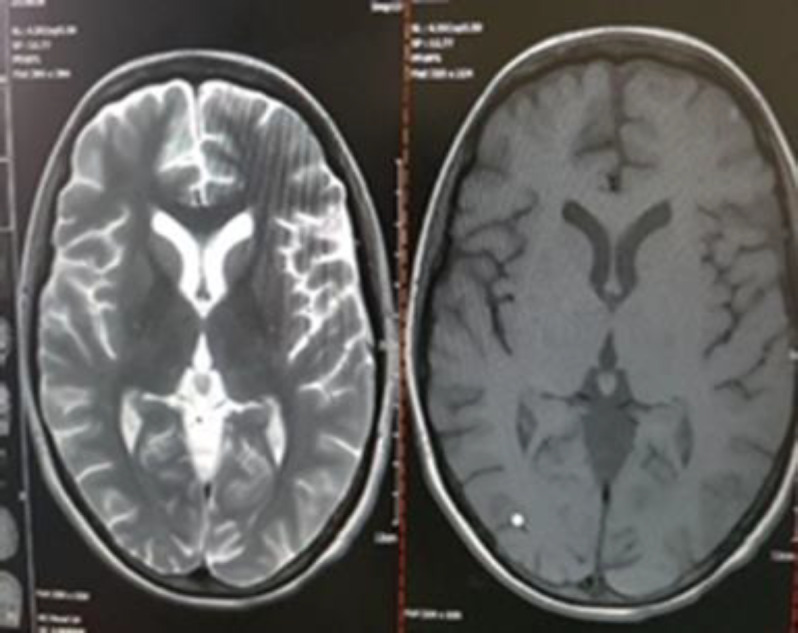
Follow-up MRI after three weeks of combination therapy and four sinus debridement procedures

During the third week of combination therapy, there was a remarkable improvement in the patient’s clinical signs and symptoms. Also, in the follow-up brain MRI images, the frontal lobe lesion was completely resolved (figure 5). The combination antifungal therapy continued for four weeks, and the patient was discharged in the twelfth week. In the weekly follow-ups, no relapse in ROCM was found during the first three months of discharge. However, after three months, the patient died because of relapse in hematologic disease and bleeding due to severe thrombocytopenia. 

## Discussion

In recent years, the number of diagnosed cases of mucormycosis has increased probably due to increased awareness of the signs and symptoms of this infection and/or improvement of diagnostic techniques (7). Early diagnosis, debridement of necrotized tissues, and prompt initiation of antifungal therapy are the cornerstones of successful treatment for invasive mucormycosis, which can reduce the associated morbidity and mortality (1). 

Although surgery is an important part of treatment for mucormycosis, some patients do not meet the essential preoperative criteria or refuse to undergo surgery (similar to our case). Generally, there is scarce information about clinical decision-making in these patients, especially when there is an intracranial invasion. Therefore, in this study, we reported our successful experience in a patient with such conditions. In our patient, a combination antifungal therapy, concomitant with multiple endoscopic sinus debridement procedures, was effective in the treatment of ophthalmic involvement and intracranial lesion, as confirmed by the follow-up MRI images.

LAMB has an excellent inhibitory concentration in the eye and brain tissues (8). Although the concentration of posaconazole in the eye and brain tissues is low, its concentration increases in inflamed tissues (8). Combination antifungal therapy with LAMB plus posaconazole has a remarkable synergistic effect on the inhibition of Mucorales species in vitro (9). One of the possible explanations for this effect is that LAMB can create membrane holes in Mucorales species and facilitate the entry of posaconazole into the cells (10). The efficacy of this regimen in patients with mucormycosis has been indicated in some previous reports (1, 11, 12).

In this regard, in a case series of 32 patients with hematologic malignancies and mucormycosis, who had received a combination of LAMB and posaconazole, favorable clinical responses were reported in 56% of patients (12). In this study, the median duration of maintenance therapy was 74 days in patients with a good therapeutic response (12). However, the mentioned study did not report the efficacy of combination therapy in the improvement of intracranial lesions without craniotomy. 

In summary, endoscopic debridement, along with a combination of LAMB and posaconazole, may be a proper therapeutic option for patients with ROCM, who are not eligible candidates for extensive surgery or craniotomy.
